# A Triad of Intricacies: An Exploration of Concomitant Codominant Coronary Artery Pattern, Patent Foramen Ovale, and Chiari Network in a Cadaveric Study

**DOI:** 10.7759/cureus.55434

**Published:** 2024-03-03

**Authors:** Sydney T Gandy, J. Scott Zimmerle, Said Maldonado, Jonathan S Lall, Chakravarthy M Sadacharan

**Affiliations:** 1 Anatomy, Tilman J. Fertitta Family College of Medicine, Houston, USA; 2 Anatomy, Tillman J. Fertitta Family College of Medicine, Houston, USA; 3 Tillman J. Fertitta Family College of Medicine, Anatomy, Houston, USA

**Keywords:** cardiac anatomy, pathologic anatomy, codominant coronary artery circulation, patent foramen ovale, coronary artery anomaly, patent foramen ovalis, cadaveric study, cadaver case report, concomitant cardiac anomalies, chiari network

## Abstract

Although findings related to codominant coronary artery circulation, patent foramen ovale (PFO), and Chiari network (CN) have been documented in isolation, there is a gap in literature detailing the unique case with the presence of all three cardiac anomalies concomitantly present in a single heart. The purpose of this case report is to detail a unique cadaveric heart case, to serve as reference to provide useful data for interventionalists and clinicians. This observational cadaveric study assessed a single donor heart obtained through the University of Houston College of Medicine’s Willed Donor Program. After meticulous dissection, relevant heart surface structures were isolated and identified. Morphometric analysis and measurements were obtained via a digital vernier caliper. The donor heart exhibited a typical codominant coronary arterial scheme, in that the posterior interventricular artery arose as a merger between the right coronary and the circumflex on the postero-inferior surface of the heart when placed in the valentine orientation. Interestingly, the antero-lateral surface of the heart was supplied via a left marginal artery (LMA) and an accessory left anterior interventricular artery.Contribution to the existing knowledge base of unique concomitant cardiac anomalies, may prove to be a beneficial future reference for interventionalists in hopes that an expanded knowledge base may lead to comprehensive and safe implementation of a wide variety of procedures.

## Introduction

The prevalence of codominant coronary artery scheme (CCAS), patent foramen ovale (PFO), and Chiari network (CN) is well-documented individually, with varying prevalence rates in the general population. The discovery of unusual concomitant cardiac abnormalities during a cadaveric observational study prompted an investigation into potential clinical complications associated with the presence of CCAS, PFO and CN.

Coronary artery branching and patterns are typically categorized into three types: right-dominant circulation, left-dominant circulation, and codominant circulation. In a codominant circulation of the heart, both the right and left coronary arteries contribute to the arterial supply of the posterior interventricular septum. Most of the current research focuses on the association between heart dominance and an increased risk of mortality following percutaneous coronary intervention, postoperative complications, difficulties during coronary artery-related interventions, and other heart-related conditions [1,2]. Current research indicates that CCAS has an approximate prevalence of 7-16% in the general population [1].

A PFO refers to an opening in the lateral septal wall that separates the right and left atria. It is a normal fetal structure designed to allow blood to bypass pulmonary circulation and enter the left atrium (LA). After birth, as pulmonary circulation is established, the PFO gradually closes due to changes in pressure between the right and left atria, typically closing between six months and one year of life. Meron et al. found that PFOs persist in about 15% to 35% of the adult population [3]. Although PFOs are relatively common, their associations with paradoxical embolisms and ischemic events are among the clinical consequences resulting from this anatomical variation. PFOs are also associated with additional anomalous cardiac findings, such as redundant interatrial septum (IAS) (atrial septal aneurysms), remnants of the sinus venosus valve (eustachian valves), and filamentous strands in the right atrium (RA) (CN) [3-5].

The CN is a fibrous/filamentous structure found within the RA, attaching to the superior wall of the RA or right atrial septal wall. This network assumes a reticular, whip-like fashion originating from the eustachian valve, connecting different parts of the RA. This incomplete resorption, however, may result in either the persistence of a prominent Eustachian valve or a CN, which shares the same point of anatomical attachment but differs in appearance. [6]. While the clinical significance of a CN is minimal, it may influence or favor PFO formation, increasing the risk of paradoxical embolism and thrombi formation. Current research indicates a CN prevalence of approximately 2% in the general population with about 80% of cases being associated with PFOs [5,6]. CNs are linked to other medical conditions, including infective endocarditis, tricuspid atresia, fetal hydrops, atrial septal hypertrophy, and atrial fibrillation [6,7]. A CN may potentially complicate interventional procedures as the fenestrated fibrous strands can trap foreign objects and cardiac catheters used in critical procedures such as atrial septal defect repair and other valvular repair procedures [7].

A comprehensive understanding of the anatomical configurations and variable presentations of coronary arteries is crucial for safe and effective cardiac procedures, including interpreting coronary angiographies, performing percutaneous coronary interventions, angioplasties, and coronary artery bypass surgeries. In perioperative and periprocedural settings, healthcare providers must be prepared for unexpected findings to minimize procedural complications or imaging challenges. In rare cases, internal cardiac pathologies and uncommon coronary artery presentations may complicate interventional procedures, presenting complex challenges for healthcare professionals. Therefore, expanding the knowledge base regarding unique concomitant cardiac anomalies can serve as a valuable future reference for interventionalists, ensuring the safe and comprehensive implementation of modified procedures.

This article was previously presented as a meeting abstract at the 2023 American Association of Clinical Anatomists (AACA) Annual Scientific Meeting on July 11, 2023.

## Case presentation

A 86-year-old woman was donated to the Tilman J. Fertitta Family College of Medicine through the Willed Donor Program. Her cause of death was attributed to breast cancer and acute respiratory failure. Gross dissection revealed the presence of a CCAS, a PFO and an extensive CN in the RA. There was no evidence of surgical innervation in the heart.

This observational cadaveric study assessed a single donor heart obtained through the Willed Donor Program. Care was taken to meticulously dissect and remove epicardium and adipose tissue to clearly identify and isolate relevant heart surface structures. No inadvertent damage was noted during the dissection process.

Using a scalpel, an incision was made along the lateral surface of the superior vena cava, beginning approximately 2 cm superior to the base of the heart. The incision extended in a superior-inferior fashion through the entire superior vena cava and inferior vena cava (IVC) vessel wall, concluding approximately 2 cm inferior to the diaphragmatic surface of the heart. This incision provided direct visualization of the internal structures within the RA.

After the dissection was completed, measurements of the left anterior descending (LAD) artery, accessory LAD artery, left marginal artery (LMA), left circumflex artery (LCx) with a digital vernier caliper (Mitutoyo digital calipers 500-172-30) having an accuracy of ± 0.02 mm. Vessel lengths were measured in their anatomical positions by using flexible wire fixed at the vessel's origin and tracing the vessel to its terminus. The wire was then straightened and measured with the vernier caliper. Thickness for these vessels were obtained. Finally, the diameter of the PFO was taken as well. All measurements and findings were reported in reference to the normal anatomical position.

The study investigated the anatomy of coronary arteries and their branches in the heart. The right coronary artery (RCA) and left coronary artery (LCA) originate from the corresponding aortic sinuses at the proximal part of the ascending aorta (AA). The RCA arises from the right aortic sinus of the AA and courses to the right side of the pulmonary trunk within the coronary sulcus. In approximately 60% of cases, the first branch of the RCA is the sino-atrial nodal branch, which supplies the sinoatrial (SA) node [8].

In this study, it was observed that the RCA gave rise to an accessory anterior interventricular artery, which is normally a branch of the LCA. The anterior interventricular branch of the LCA descends within the anterior interventricular groove and is accompanied by the great cardiac vein. This artery usually reaches the apex, but it may extend beyond the apex to the posterior (inferior) aspect of the heart. The anterior interventricular branch supplies adjacent parts of the right and left ventricles and the anterior two-thirds of the interventricular septum. In many cases, the anterior interventricular branch gives rise to a diagonal branch, which descends to the anterior surface of the heart [8].

The RCA gives rise to the right marginal branch, providing blood supply to the right border of the heart without extending to the apex. Towards the posterior aspect, the RCA typically issues the atrioventricular nodal branch in 80% of cases, supplying the atrioventricular node [8]. The RCA then branches as the posterior right ventricle artery, contributing to the blood supply of the right ventricle. Eventually, in our study, the RCA joins the LCA and terminates as the posterior descending artery (PDA).

The LCA originates from the left aortic sinus, coursing between the left auricle and the left side of the pulmonary trunk. In approximately 40% of cases, the circumflex branch of the LCA gives rise to the SA nodal branch, directing towards the posterior surface of the LA and supplying the SA node [8]. In the left coronary sulcus nearing the posterior side of the heart, the circumflex branch bifurcates into the LMA and the PDA. The posterior interventricular branch descends posteriorly and inferiorly toward the apex. 

**Figure 1:**
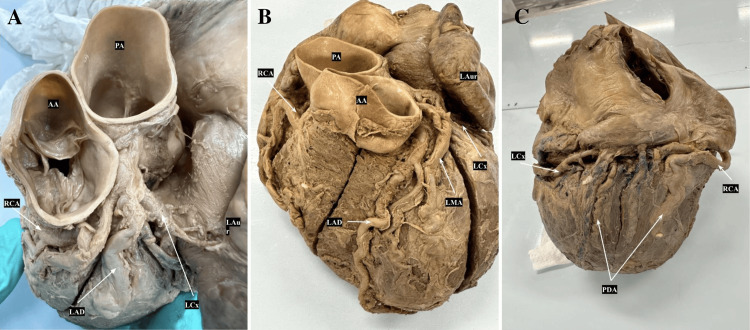
Coronary Artery A: Anterosuperior view of the heart illustrating coronary artery branching pattern. B: Anterior overview of the heart illustrating vasculature patterns C: Posterior view of the heart illustrating codominate PDA branching pattern AA: Ascending Aorta; RCA: Right Coronary Artery; LCx: Left Circumflex Artery; LMA: Left Marginal Artery; LAD: Left Anterior Descending; LAur: Left Auricle; LB: Limbus; PA: Pulmonary Artery; PDA: Posterior Descending Artery

The arterial supply is highlighted in Figure 1. The detailed measurements of lengths, thickness, and distances of coronary arteries and their branches contribute valuable insights into the intricate vascular supply of distinct heart regions and are recorded in Table 1.

**Table 1:** Measurement of the Major Cardiac Arteries LAD: Left Anterior Descending; LMA: Left Marginal Artery; RCA: Right Coronary Artery

	Length	Thickness	Distance
LAD Artery	108.36 mm	3.99 mm at Origin; 3.32 mm at the Apex	9.6 mm from Trunk
Accessory LAD Artery	86.85 mm	2.42 mm at Origin; 0.75 mm at the Apex	8.9 mm from Trunk
LMA	50.82 mm	2.58 mm at Origin	4.83 mm from Trunk
Circumflex Artery	132.57 mm	4.29 mm at Origin; 1.91 mm at Terminal Point	-
Artery of the Posterior Right Ventricle from RCA	-	3.75 mm at Origin; 1.79 mm at Terminal Point	-

**Figure 2:**
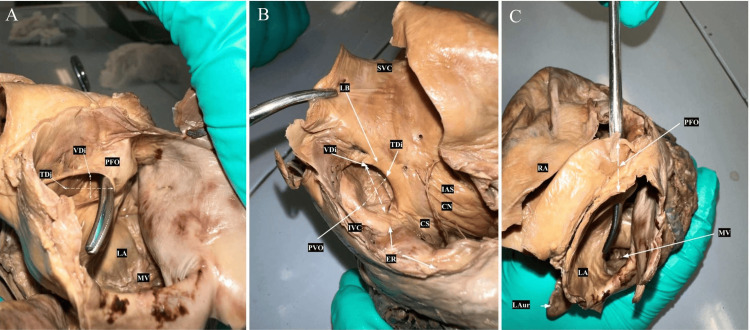
Foramen Ovale A: Superolateral view looking into the RA. Major landmarks are labeled. B: Superior view displaying a probe passing through the PFO shunt within the RA and emerging through its orifice in the LA C: Superior Anterolateral view of the LA displaying the approximate plane of measurements for the vertical diameter and the transverse diameter. CN: Chiari Network; ER: Eustachian Ridge; IAS: Interatrial Septum; IVC: Inferior Vena Cava; LAur: Left Auricle; LA: Left Atrium; LB: Limbus; MV: Mitral Valve; PFO: Patent Foramen Ovale; PFOSh: Patent Foramen Ovale Shunt; RA: Right Atrium; SVC: Superior Vena Cava; TDi: Transverse Diameter; VDi: Vertical Diameter

In our investigation, we noted a slight inferior displacement of the foramen ovale (FO) towards the opening of the IVC on the IAS, as illustrated in Figure 2. The FO displayed an elliptical shape, with an average transverse diameter of 24.21 mm (range, 12-26 mm) and an average vertical diameter of 26.84 mm (range: 14-28 mm). The most pronounced limbal prominence was found at the inferior and posterior margins, with a thickness of 0.84 mm. The FO's floor exhibited a thickness of 11.19 mm.

We examined the patency of the FO through visual inspection and probe assessment, revealing a slit-like appearance along the anterior aspect of the annulus, as depicted in Figure 2a and Figure 2b. The FO's dimensions were measured in both the RA and LA. In the RA, the FO had a transverse diameter of 10.56 mm and a vertical diameter of 22.16 mm, while in the LA, it measured 14.18 mm in transverse diameter and 19.83 mm in vertical diameter as shown in Figure 2c.

**Figure 3:**
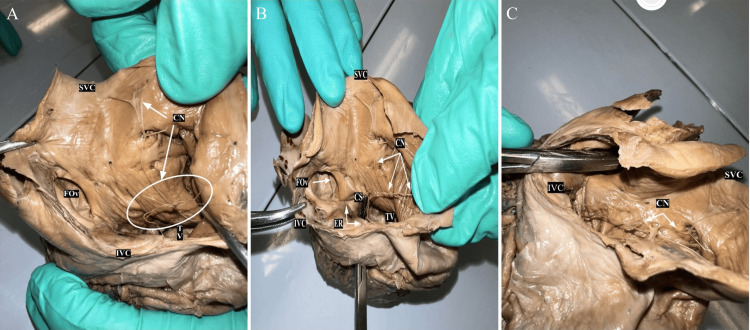
Chiari Network A: Interior of RA showing the opening of the FO, ER, and the CN are indicated by white arrows B: Superior-Inferior View of the RA showing the CN. A probe is traversing the IVC (white arrows). The CN is the filamentous structure traversing the RA. C: The CN is shown with fine strands connected to the walls of the vena cava. The network overlays the IVC lateral to the entrance to the FO. CN: Chiari Network; CS: Coronary Sinus; ER: Eustachian Ridge; FO: Foramen Ovale; IVC: Inferior Vena Cava; RA: Right Atrium; SVC: Superior Vena Cava; TV: Tricuspid Valve

During our dissection, we repositioned the walls of the IVC and superior vena cava (SVC) to gain access to the contents of the RA. The CN in our specimen exhibited a fine reticular network with a few membranous structures attached to the walls of the RA, as visualized in Figure 3. These slender thread-like strands extended in a whip-like fashion along much of the vena cava, with the membranous portion primarily flat. The principal attachment site of the CN was situated along the Eustachian ridge (ER), extending to the RA's wall, as depicted in Figure 3. In this specimen, the CN covered the IVC and the entrance of the FO. In summary, our study provides meticulous measurements and observations of the FO and the CNs in this specific specimen.

## Discussion

Cardiac dominance, determined by the artery from which the posterior interventricular artery or PDA branches, has three types: right dominant (PDA branches from the RCA), left dominant (PDA branches from the LCx), and codominant (PDA results from the merger of the RCA and LCx). Right-dominant hearts are the most common (70-80%), while CCAS hearts are less common (10-20%) [9]. Various genes and progenitor cells influence coronary artery branching patterns, including Vegf-A, Vegfr1, Vegfr2, and Jagged [10]. Awareness of coronary circulation variations is crucial for interventional cardiologists, congenital heart surgeons, and interventional radiologists. Identifying anomalous branching patterns can be challenging even with advanced imaging techniques. Left-dominant hearts have higher mortality rates in cases of acute coronary syndrome compared to right-dominant hearts, while co-dominant and right-dominant hearts exhibit similar mortality rates [2]. Left-dominant hearts also have an increased risk of atherosclerotic plaque formation and myocardial infarction, likely due to the complex coronary branching patterns [11]. Cardiac dominance can affect myocardial perfusion imaging (MPI) studies, with non-right dominant hearts showing an increased prevalence of false-positive stress single-photon emission computer tomography (SPECT) MPI results, particularly in the inferior or inferolateral regions [12]. The SPECT is a non-invasive procedure that can accurately identify areas of abnormal myocardial perfusion, determine the functional capacity of your heart muscle, and separate viable (living) from non-viable (irreversibly damaged) tissue. A nuclear stress test, also referred to as a MPI study, is a type of stress test that uses an imaging contrast agent known as a radiotracer to take pictures of the heart during stress and rest conditions. A PET or SPECT camera is used for imaging of the heart. Conventional SPECT MPI evaluates the presence, extent, and degree of myocardial ischemia or infarction (i.e., flow-limiting defects).

Fetal circulation differs significantly from that of adults, as oxygenated blood is delivered exclusively through the umbilical vein from the placenta. Within the fetus, blood bypasses the nonfunctional pulmonary circuit through various shunts, starting with the ductus venosus in the liver. From the ductus venosus, blood moves through the IVC and enters the right atrium, where it passes through the FO, a one-way valve that allows oxygenated blood to flow into the LA before entering the left ventricle and then the primitive systemic circulation. The formation of the FO involves the septum primum and secundum, which develop and fuse to create a valve that closes off the foramen primum and initiates atrial septation. Normally, at birth, the septum primum and secundum fuse due to changes in pressure as the pulmonary arteries open and the alveoli fill with oxygen, along with increased left atrial pressure from pulmonary blood flow. This forces the valve to close. Although many patients with a PFO are asymptomatic, it has been associated with an increased risk of cardiovascular events, including paradoxical emboli and strokes. The prevalence of PFO in adults is estimated at 20-25%, but it rises to 40-50% in patients with cryptogenic strokes [13]. Patients with cryptogenic strokes who receive medical therapy alone have a significantly higher risk of recurrent neurological events compared to those who undergo PFO closure [14,15]. The Randomized Evaluation of Recurrent Stroke Comparing PFO Closure to Established Current Standard of Care Treatment (RESPECT) trial, revealed that the risk reduction was more significant in patients with extended follow-up. PFOs have also been linked to migraines, with the Prospective, Randomized Investigation to Evaluate Incidence of Headache Reduction in Subjects With Migraine and PFO Using the AMPLATZER PFO Occluder to Medical Management (PREMIUM) trial showing “subjects in the PFO closure group had a significantly greater reduction in headache” and “8.5% of patients experienced a complete remission of migraine over a 1-year time period” [15]. The PREMIUM was a double-blind study investigating migraine characteristics over one year in subjects randomized to medical therapy with a sham procedure (right-heart catheterization) versus medical therapy and PFO closure with the AMPLATZER PFO Occluder device (St. Jude Medical, St. Paul, USA). Our examination revealed not only significantly large dimensions of the PFO but also an inferior displacement. These observations contribute to our current understanding of fetal circulation patterns and with PFOs associated increased risk of cardiac events, underscores the importance of recognizing and understanding the variations identified in our study.

CNs result from variations in normal developmental anatomy. Originally, the presence of a CN was attributed to the incomplete resorption of the right valve of the sinus venosus, and sometimes the septum spurium. Initially described by Carl von Rokitansky in 1875 and further detailed by Hans Chiari in 1897, a single case was described as a fibrous mesh of tissue attaching to the crista terminalis, the ostium of the IVC, the tubercle of Lower, and the Eustachian valve. However, recent reviews and studies have shown that CNs can vary in their position and orientation within the right atrium. This variability in presentation has led to different interpretations of their embryological and developmental origins, including the possible involvement of the left valve of the sinus venosus [6]. In normal heart development, the right and left valves of the sinus venosus fenestrate as they resorb into the interatrial septal wall, leaving no remnant. Incomplete resorption, depending on the extent of fenestration, can lead to the formation of reticular webs of tissue in the case of the right valve or a trabecular region cranial to the fossa ovalis in the case of the left valve's failure to resorb. In this case, the heart displayed a CN originating from the inferolateral wall of the right atrium, just superior to the Eustachian valve. The primary network did not extend outward from the atrial wall, but there were numerous projections of the network reaching the medial wall of the right atrium, with some attaching anterior to the PFO.

The clinical significance of CN remains uncertain but adds a layer of complexity to our discussion. Prominent CN, distributed over a large portion of the right atrium, have been known to interfere with guide wires, pacemaker leads, and pose challenges for various interventional cardiology procedures. Flow disturbance and impedance associated with prominent CNs may promote thrombus formation, potentially increasing the risk of paradoxical emboli and strokes, especially since patients with CNs are more likely to have a PFO [6]. Additionally, in patients without a PFO, there may be a higher risk of pulmonary embolism due to increased turbulence caused by the CN. However, some experts suggest that fenestrated CN variations may act as a biological filter for blood flow through the IVC into the right atrium, potentially preventing large pulmonary embolisms as the fenestrations can trap existing emboli. Prominent CN have also been mistakenly diagnosed as atrial masses, valve vegetations, or thrombi on imaging via transesophageal echocardiography (TEE) and transthoracic echocardiography (TTE) due to the lack of contrast heterogeneity within the right atrium [6]. Manerikar et al. described a case in which a patient underwent a minimally invasive open-heart procedure to remove a 1.3 cm mitral valve (MV) mass but instead found a prominent CN [4].

## Conclusions

This study delves into the examination of various anatomical variations and congenital abnormalities within the heart of a single cadaver. Previously studied in isolation, the abnormalities, including codominant arterial supply, PFO, and extensive CNs, are now explored collectively.The cadaveric case study sheds light on the intricate interplay of anatomical variations in the cardiovascular system. The coexistence of these abnormalities in a single donor heart is statistically rare, and the observed variations in branching patterns, PFO dimensions, and the extent of CN involvement offer crucial insights into the complexity of heart anatomy. The presence of these anomalies underscores the importance of awareness among healthcare professionals, particularly in interventional cardiology and congenital heart surgery. Recognizing and understanding these variations can significantly contribute to accurate diagnoses, guide procedural approaches, and reduce complications. Future exploration of each anomaly in isolation and concurrently will further enhance our comprehension of their clinical significance, paving the way for tailored interventions, improved patient outcomes, and advancements in cardiac healthcare.
